# Tissue Distribution of ACE2 Protein in Syrian Golden Hamster (*Mesocricetus auratus*) and Its Possible Implications in SARS-CoV-2 Related Studies

**DOI:** 10.3389/fphar.2020.579330

**Published:** 2021-01-14

**Authors:** Voddu Suresh, Deepti Parida, Aliva P. Minz, Manisha Sethi, Bhabani S. Sahoo, Shantibhusan Senapati

**Affiliations:** ^1^ Tumor Microenvironment and Animal Models Lab, Institute of Life Sciences, Bhubaneswar, India; ^2^ Regional Centre for Biotechnology, Faridabad, India; ^3^ Institute of Life Sciences, Bhubaneswar, India

**Keywords:** lung, COVID 19, angiotensin converting enzyme-2, SARS CoV-2, hamster (Mesocricetus auratus)

## Abstract

The Syrian golden hamster (*Mesocricetus auratus*) has recently been demonstrated as a clinically relevant animal model for SARS-CoV-2 infection. However, lack of knowledge about the tissue-specific expression pattern of various proteins in these animals and the unavailability of reagents like antibodies against this species hampers these models’ optimal use. The major objective of our current study was to analyze the tissue-specific expression pattern of angiotensin-converting enzyme 2, a proven functional receptor for SARS-CoV-2 in different organs of the hamster. Using two different antibodies (MA5-32307 and AF933), we have conducted immunoblotting, immunohistochemistry, and immunofluorescence analysis to evaluate the ACE2 expression in different tissues of the hamster. Further, at the mRNA level, the expression of *Ace2* in tissues was evaluated through RT-qPCR analysis. Both the antibodies detected expression of ACE2 in kidney, small intestine, tongue, and liver. Epithelium of proximal tubules of kidney and surface epithelium of ileum expresses a very high amount of this protein. Surprisingly, analysis of stained tissue sections showed no detectable expression of ACE2 in the lung or tracheal epithelial cells. Similarly, all parts of the large intestine were negative for ACE2 expression. Analysis of tissues from different age groups and sex didn’t show any obvious difference in ACE2 expression pattern or level. Together, our findings corroborate some of the earlier reports related to ACE2 expression patterns in human tissues and contradict others. We believe that this study’s findings have provided evidence that demands further investigation to understand the predominant respiratory pathology of SARS-CoV-2 infection and disease.

## Introduction

The current outbreak of COVID-19 (Corona Virus Disease 2019) caused by the SARS-CoV-2 virus was declared a pandemic on March 11, 2020. It has now succumbed to around 44 million people worldwide, and almost 1.17 million people have lost their lives due to this pandemic (till October 28, 2020). Therefore, there is an urgent need to study this viral disease transmission, pathogenesis, prevention, and treatment. In this regard, the role of clinically relevant experimental animal models is crucial. No single species of animal might be able to recapitulate all the SARS-CoV-2 infection-related events in humans exactly. However, using different animal models will help to address questions in a more reliable and clinically relevant manner. Simultaneously, exploring multiple species susceptible to this virus might also help to identify the natural reservoir and potential carriers of this pathogen.

Syrian golden hamsters (*Mesocricetus auratus*) being a permissive host to multiple other viruses and a recognized model for respiratory syndrome coronavirus (SARS-CoV) infection has drawn immediate attention for COVID-19 related studies (Roberts et al., 2005). Studies have shown that the Syrian hamster infected by SARS-CoV-2 manifest various clinical signs of COVID-19 in human patients (Chan et al., 2020a; Chan et al., 2020b; Sia et al., 2020). Moreover, the pathology of this disease in hamsters resembles humans. The model has also demonstrated high transmissibility of SARS-CoV-2 among close contact animals. In a recent study, male Syrian golden hamsters at 4–5 weeks of age were used to check the potential of hamster as a model of SARS-CoV-2 infection (Sia et al., 2020). Analysis of tissue samples from the infected animals showed pathological changes in the respiratory tract and SARS-CoV-2 N protein at bronchial epithelial cells, pneumocytes, and nasal epithelial cells. Duodenum epithelial cells also stained positive for viral N protein. No apparent histopathological changes were observed in the brain, heart, liver, and kidney on five dpi (days post-infection) (Sia et al., 2020). Although no infectious virus was detected in the kidney, low copies of the viral genome were detected on two and five dpi (Sia et al., 2020). In a similar type of study, by using male and female Syrian hamsters of 6–10 weeks old, identified tissue damage and viral N protein presence at different parts of the respiratory tract (nasal turbinate, trachea, and lungs) (Chan et al., 2020a). The viral N protein was abundantly present in bronchial epithelial cells, macrophages, type I, and II pneumocytes. At four dpi, N protein expression was found all over the alveolar wall (Chan et al., 2020a). The histopathological analysis also showed tissue damage and/or inflammatory lesions at multiple extra-pulmonary organs (intestine, heart, spleen, bronchial, and mesenteric lymph nodes); however, N protein expression was only detected in the intestinal epithelial cells (Chan et al., 2020a). In the very recent past, the Syrian hamster model of SARS-CoV-2 infection has been instrumental in establishing that passive transfer of a neutralizing antibody (nAb) protects SARS-CoV-2 infection (Rogers et al., 2020).

Studies have clearly shown SARS-CoV-2 binds to human angiotensin-converting enzyme 2 (ACE2) expressed by its target cells and use it as a functional receptor to enter into cells (Hoffmann et al., 2020; Walls et al., 2020). Hence, drugs that could inhibit the binding of viral proteins (S-protein) to the ACE2 expressed on the target cells are assumed to be potential therapeutics against COVID-19. A recent study has shown that human recombinant soluble ACE2 (hrsACE2) blocks the early stage of SARS-CoV-2 infection (Monteil et al., 2020). We have also proposed the bioengineered probiotics expressing human ACE2 as a potential therapeutics against SARS-CoV-2 infection (Senapati et al., 2020a). The ACE2 protein’s sequence alignment of different species has suggested that the S protein may interact more efficiently with Cricetidae ACE2 than murine ACE2 (Luan et al., 2020). *In silico* analysis also shows possible interaction between SARS-CoV-2 spike proteins with Syrian hamster ACE2 (Chan et al., 2020a).

At the time of the ongoing COVID-19 pandemic, in addition to the vaccine and antiviral development, attempts have been made to target host proteins for therapeutic purposes. As discussed above, the pharmaceutical modulation of ACE2 expression or inhibition of its interaction with SARS-CoV-2 spike protein for COVID-19 therapy is a matter of current investigation at different parts of the world (Kai and Kai, 2020). In these efforts, animal models will be instrumental in checking potential drug candidates’ efficacies and safety against COVID-19. Although the Syrian hamster is a clinically relevant model for multiple infectious diseases, the unavailability of reagents like antibodies against hamster proteins and lack of publicly available gene or protein expression data for this species are the major constraints to using these models up to their full capacity (Suresh et al., 2019). Before utilizing hamster as a model to understand the role of ACE2 in the pathogenesis of SARS-CoV-2 infection and/or to evaluate the efficacy of ACE2-targeted drugs, knowledge about the basal level of ACE2 expression in different tissues of hamster is essential. In the current study, we have checked the expression pattern of ACE2 in different tissues of normal Syrian hamsters through immunoblotting, immunohistochemistry, and immunofluorescence staining techniques.

## Material and Methods:

### Isolation of Hamster Tissue Samples

The tissue samples used for initial antibody standardization are from archived samples collected during our previous studies (Suklabaidya et al., 2016; Suresh et al., 2019). To analyze ACE2 expression in hamsters of different age groups and sexes, a separate Institutional Animal Ethical Committee (Institute of Life Sciences, Bhubaneswar, India) approval was obtained before conducting the study (Project no.: - ILS/IAEC-195-AH/Jul-20). All the methods associated with animal studies were performed according to the Committee for the Purpose of Control and Supervision of Experiments on Animal (CPCSEA), India guidelines. Three age groups of animals comprising of young (∼2–4 months old), adult (∼6–8 months old), and old (∼15–17 months old) were included in this study. For each age group, organs from six different animals (three males and three females) were harvested and preserved for further processing and analysis.

### Western Blot Analysis

Using an electric homogenizer, tissues were lyzed in ice-cold RIPA buffer (20 mM Tris-HCl pH 7.5, 150 mM NaCl, 1 mM Na2 EDTA, 1 mM EGTA, 1% NP-40, 1% sodium de-oxy-cholate, 2.5 mM sodium pyrophosphate, 1 mM β-glycerophosphate, 1 mM Na3VO4) supplemented with a protease inhibitor cocktail (MP Biomedicals) and soluble proteins were collected. Protein concentrations were measured by Bradford assay (Sigma). 20 µg of protein was loaded for each sample and electrophoresed through 8% SDS-polyacrylamide gels. Proteins were transferred to poly-vinylidene difluoride membrane (Millipore) and blocked with 5% bovine serum albumin. Membranes were probed with ACE2 (#MA5-32307; Invitrogen; 1:3000 or #AF933; R&D Systems: 1 μg/ml) or β-actin (#A2066; Sigma-Aldrich; 1:1,000) primary antibody and horseradish peroxidase-conjugated secondary antibody. Antibody binding was detected with electrochemiluminescence substrate (#12757P; CST) and chemiluminescence visualized with ChemiDoc™MP Gel Imaging System (BioRad).

### Immunohistochemistry

All the tissue samples were processed and sectioned as reported earlier (Suklabaidya et al., 2016; Suresh et al., 2019). Paraffin-embedded sections were de-paraffinized using xylene, rehydrated in graded ethanol, and deionized water. Sections were subjected to antigen retrieval treatment by boiling in acidic pH citrate buffer (Vector Laboratories) for 20 min in a steam cooker. 3% hydrogen peroxide in methanol was used to block the endogenous peroxidase for 20 min and washed with 1X PBS two times, followed by blocking with horse serum (Vector Lab) for 30 min at room temperature. Sections were treated with ACE2 antibody (#MA5-32307, 1:200 or #AF933; 2 μg/ml) overnight in a humidified chamber at 4°C. Sections were washed twice with 1X PBS for 5 min each. In MA5-32307 antibody case, slides were treated with horse anti-rabbit/mouse IgG biotinylated universal antibody (Vector Laboratories) for 45 min at room temperature and with ABC reagent for 30 min. For AF933 antibody, the slides were incubated with Goat IgG VisUCyte HRP Polymer (#VC004, R&D Systems) and incubated for 45 min at room temperature (without ABC incubation). To develop the stain 3, 3′-diaminobenzidine (DAB; Vector Laboratories) was used as a substrate according to the manufacturer’s instructions, and hematoxylin was used as a counter-stain. Sections were dehydrated with ethanol, cleared with xylene, and mounted with Vecta mount permanent mounting medium. Sections were observed under the microscope (Leica ICC500), and images were captured at ×40 magnification.

### Immunofluorescence

Paraffin sections were subjected to de-paraffinization, rehydration, and antigen retrieval treatment. Sections were blocked with horse serum (Vector Lab) for 30 min at room temperature and probed with ACE2 primary antibody (#MA5-32307, 1:200 or #AF933; 2 μg/ml) overnight in a humidified chamber at 4°C. Sections were washed twice with PBST and treated with anti-Rabbit Alexa Fluor 594 (#A-11037; Life technologies, 1:500) or anti-Goat Alexa Fluor 594 (#A-11080; Life technologies, 1:500) at room temperature for 45 min under dark conditions. After washing, slides were mounted with SlowFade Gold Antifade mountant with DAPI (#S36938; Life Technologies) and visualized using Leica TCS SP8STED confocal microscope.

### Antigen Preadsorption Test

Antibodies (#MA5-32307 or #AF933) were pre-incubated with human recombinant ACE2 protein (#933-ZN; R&D systems) at 1:5 weight to weight ratio or antibodies alone in microcentrifuge tubes containing 1% BSA. Incubation was performed by keeping the tubes with slow rotations overnight at 4°C. After the incubation, antibodies were used for IHC and IF staining of selected hamster tissues.

### Quantitative Real-Time PCR

Tissues from animals were harvested for RNA isolation following the Trizol method using TRIzol^®^ LS Reagent (#10296028; Thermo). Synthesis of cDNA was performed using High Capacity cDNA Reverse Transcription Kit (#4368814; Applied Biosystems), and qRT-PCR was performed using the BRYT green PCR master mix (#A6002; Promega) according to the manufacturer’s protocol. *Gapdh* or *β-actin* was used for normalization. The expression status of *Ace2* was analyzed using the following specific primers: forward: 5′- GAA​GAG​GCT​GTC​AGG​TTG​TC -3′ and reverse 5′- TGC​CAA​CCA​CTA​CAA​TTC​CC -3'.

## Results and Discussions

This study’s major objective was to check the status of ACE2 protein expression in hamster tissues of different age groups. Traditionally, immunohistochemistry or immunocytochemistry are widely used techniques to localize specific proteins or epitopes in cells and tissues. The authenticity of interpretations based on these techniques requires multiple controls in an application- and context-dependent manner. The International Working Group for Antibody Validation (IWGAV) has suggested five different pillars for antibody validation. A minimum of one pillar should be used to validate an antibody’s suitability for a particular application (Uhlen et al., 2016). To get a convincing and reliable interpretation of our study, we have adopted multiple controls, which includes correlation between *Ace2* mRNA and protein expression in tissues (orthogonal strategy), use of two different antibodies and comparison of staining pattern (independent antibody strategy), reactivity toward purified human recombinant ACE2 protein (rHu-ACE2), and antigen preadsorption test. We have also conducted both IHC and IF staining for most of the organs.

**FIGURE 1:**
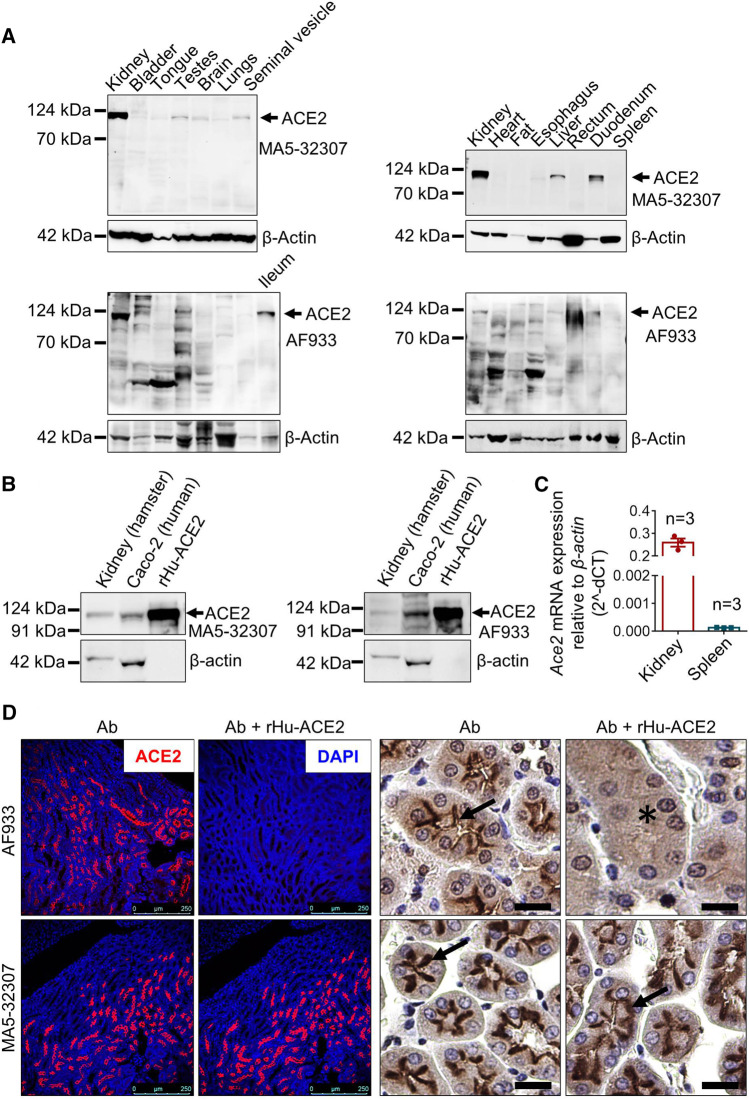
Validation of MA5-32307 and AF933 antibodies’ reactivity and specificity toward hamster ACE2. **(A)** Immunoblot analysis showing the expression status of ACE2 with two different antibodies (MA5-32307 and AF933) in multiple organ tissues of the Syrian golden hamster. β-actin was used as an internal control. **(B)** Immunoblots showing ACE2 expression in hamster kidney, human colorectal adenocarcinoma cells (Caco2), and recombinant human ACE-2 protein (rHu-ACE2) with two different antibodies. **(C)** Graph showing *Ace2* relative mRNA expression in hamster kidney and spleen tissues. **(D)** Micrographs showing immunofluorescence and immunohistochemical staining of hamster kidney with antibody alone or antibody preincubated with rHu-ACE2 protein (in IHC, scale bar = 25 µm).

In this study, the ACE2 protein expression was evaluated by two different antibodies. The ACE2 recombinant rabbit monoclonal antibody (Invitrogen; clone SN0754; Cat No MA5-32307) used in this study was generated by using synthetic peptide within human ACE2 aa 200–230 as an immunogen. As per the information available by different companies, this clone (SN0754) has reactivity against human, mouse, rat, and hamster (Invitrogen: #MA5-32307 and Novus Biologicals: #NBP2-67692). The polyclonal goat IgG (R&D Systems; Cat No AF933) was generated by using mouse myeloma cell line NS0-derived human recombinant ACE-2 (Gln18-Ser740; Accession# Q9BYF1). The information available with the product indicates its reactivity against human, mouse, rat, and hamster proteins. There are 26 species of hamsters in the world. In the recent past Syrian golden hamster (*Mesocricetus auratus*) has been demonstrated as a clinically relevant model for SARS-CoV-2 infection (Chan et al., 2020a; Sia et al., 2020; Chan et al., 2020b). Hence, to check whether these antibodies have reactivity against the Syrian golden hamster ACE2, we initially did an immunoblot analysis of proteins isolated from these animals’ different tissues. The antibodies showed clear reactivity with a protein of molecular weight of ∼120 kDa, which matches with ACE2 (Figure 1A). The protein lysates used in this experiment were harvested from an adult male hamster. Although similar conditions were adapted for both the antibodies, AF933 showed more nonspecific bands than MA5-32307, which could be due to the polyclonal nature of AF933. To further confirm these antibodies’ reactivity against hamster and human ACE2, we carried out immunoblot analysis of samples, including hamster kidney lysates, Caco2 human colon cancer cells lysate (known to express ACE2) (Wang et al., 2020), and rHu-ACE2. The data shown in Figure 1B confirms the reactivity of both the antibodies against human and hamster ACE2.

The immunoblot analysis results showed a high amount of ACE2 expression in hamster kidney tissues and almost no expression in spleen tissues (Figure 1A). We adapted an orthogonal strategy to further validate the antibodies by measuring mRNA expression in these two tissues. The qPCR analysis results showed a high level of *Ace2* expression in kidney tissues and minimal expression in the spleen (Figure 1C), matching with the immunoblot results. Together, these findings validate the reactivity of both the antibodies against hamster ACE2.

In experimental conditions, proteins are generally in the near-native form in the absence of denaturing agents; however, they are partly or fully denatured during western blotting or immunohistochemistry. Detection of human recombinant ACE2 protein by both the antibodies in the western blotting indicates the reactivity of these antibodies against the corresponding epitopes present in ACE2 proteins. On the other hand, successful neutralization/blocking of AF933 by preadsorption with rHu-ACE2 (Cat No 933-ZN; actual immunogen used to generate this antibody), but failure with MA5-32307 in IHC and IF staining (Figure 1D and Supplementary Figure S1A) indicates possible inaccessibility of MA5-32307-specific epitope in native rHu-ACE2 protein. During the antibody and immunogen incubation/preadsorption step, no denaturing agents were present; hence, it is highly expected that rHu-ACE2 was in its native form at this step. Although the peptide used for the generation of MA5-32307 Ab (ACE2 aa 200–230) is present in the rHu-ACE2 protein, its possible non-surface localization might have led to unsuccessful blocking of antigen-binding sites of the antibody. At the same time, a similar type of cell and region-specific staining by both the antibodies in kidney tissues (Figure 1D) indicates that tissue fixation with formalin and further downstream processing might have exposed the MA5-32307-reactive epitopes present on the target protein (ACE2). At present, ACE2 aa 200–230 peptides’ unavailability has restricted us from doing antigen preadsorption with the actual immunogen for the MA5-32307 antibody. However, a comparable pattern of reactivity of both the antibodies in detecting ACE2 protein by western blotting, IHC and IF staining strongly validate the reactivity of both the antibodies against hamster ACE2 (Figures 1A,B,D). Moreover, positive staining with MA5-32307 Ab in organs known to express ACE2 (e.g., kidney or ileum) and absence in organs known to have no or minimal expression (e.g., spleen or caecum) of other species has further validated its specificity toward hamster ACE2 (Figure 1D and Supplementary Figure S1B) (Hashimoto et al., 2012).

In our study, irrespective of antibodies, age, and sex of animals, kidney tissues showed a very high ACE2 expression (Figures 1A,B,D). It’s expression was mostly at the apical surface of proximal tubules, whereas glomeruli were negative (Figure 1D). In addition to the expected membrane staining, AF933 Ab showed some level of nuclear and cytoplasmic staining in IHC, which did not get abolished after antibody preadsorption with the immunogen (Figure 1D). Hence, the nuclear staining seen with AF933 in certain tissues, including kidney, might be due to its nonspecific reactivity toward some unknown nuclear protein(s). So far, most of the literature and publicly available protein expression database have clearly shown high expression of ACE2 protein in human kidney tissues (Monteil et al., 2020; Zhang and Zhang, 2020). High expression of ACE2 in the kidney is believed to contribute to SARS-CoV-2 virus pathogenesis and disease severity (Zhang and Zhang, 2020). Detection of kidney injuries in tissues of COVID-19 patients’ post-mortem tissues further supports the importance of considering kidney function-related issues for COVID-19 treatment and management (Diao et al., 2020). Detection of the SARS-CoV-2 viral genome in certain patients’ urine samples has also been reported (Ling et al., 2020). Using a kidney organoid model, Monteil V *et al.* have demonstrated that the proximal tubules express ACE2 and SARS-CoV-2 replicated in these organoids (Monteil et al., 2020). Despite this clinical and experimental evidence from human patients or tissues, so far, none of the SARS-CoV-2-related studies in hamsters have reported any kidney-related histopathological changes (Chan et al., 2020a; Chan et al., 2020b; Sia et al., 2020). In the future, further investigation and analysis are required to confirm whether hamster kidney epithelial cells are permissible for this viral infection. The tropism of the virus to different organs depends on multiple factors, including the organ-specific microenvironment. Simultaneously, the infectivity of isolates from different parts of the world is yet to be experimentally compared. In the future, studies with different isolates and/or hamsters with different comorbidity conditions might help to decipher the role of kidney cells in SARS- CoV- two pathogenesis.

**FIGURE 2:**
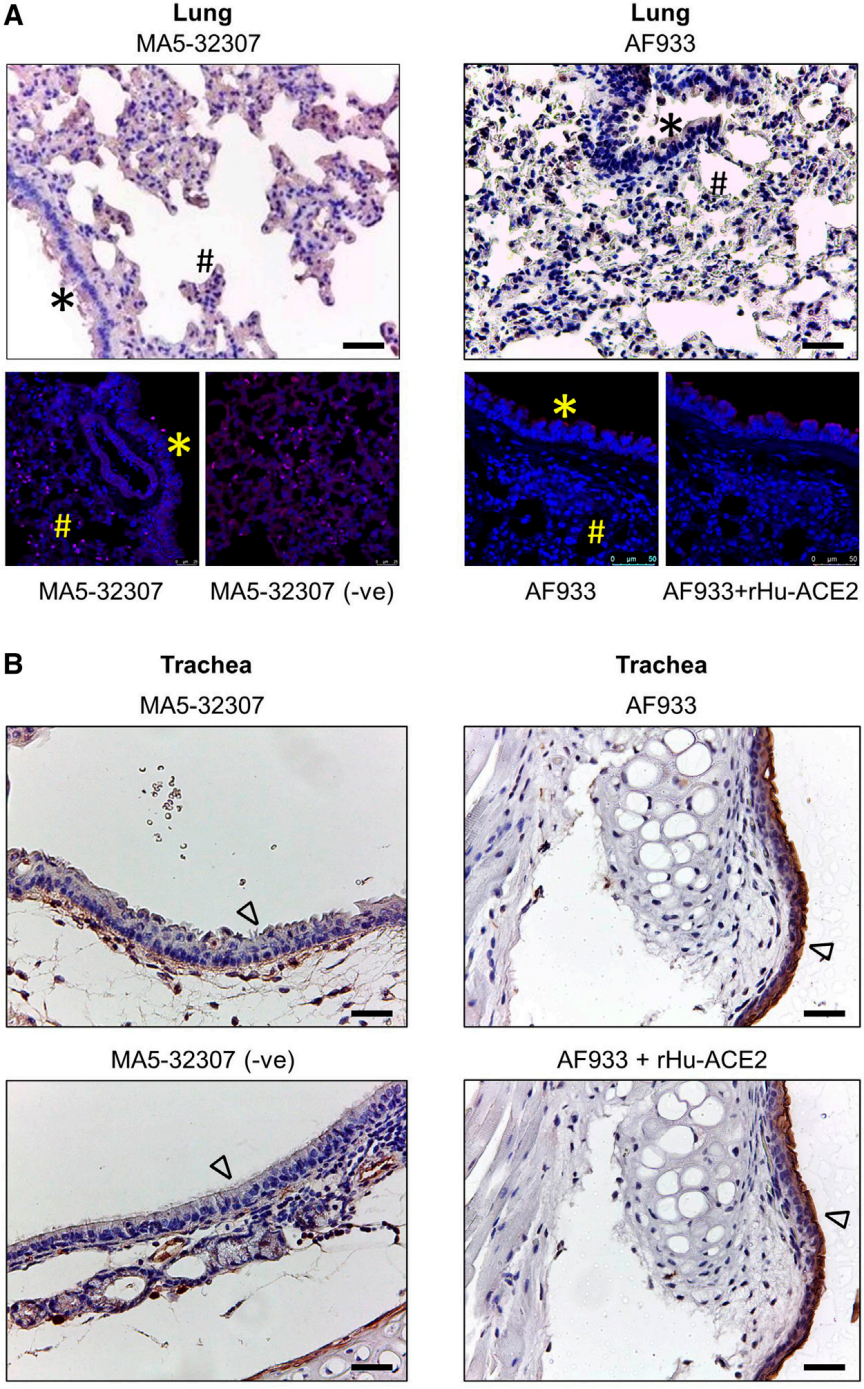
ACE2 expression pattern in hamster lung and trachea tissues. **(A**,**B)** Micrographs showing immunohistochemical staining of hamster lung and trachea tissues for ACE2. Corresponding tissues stained without primary antibody or antibody with immunogen preadsorption were used as negative controls. Cells marked with *, #, and ∆ symbols indicate bronchial, alveolar, and tracheal epithelial cells (scale bar = 50 µm).

**FIGURE 3:**
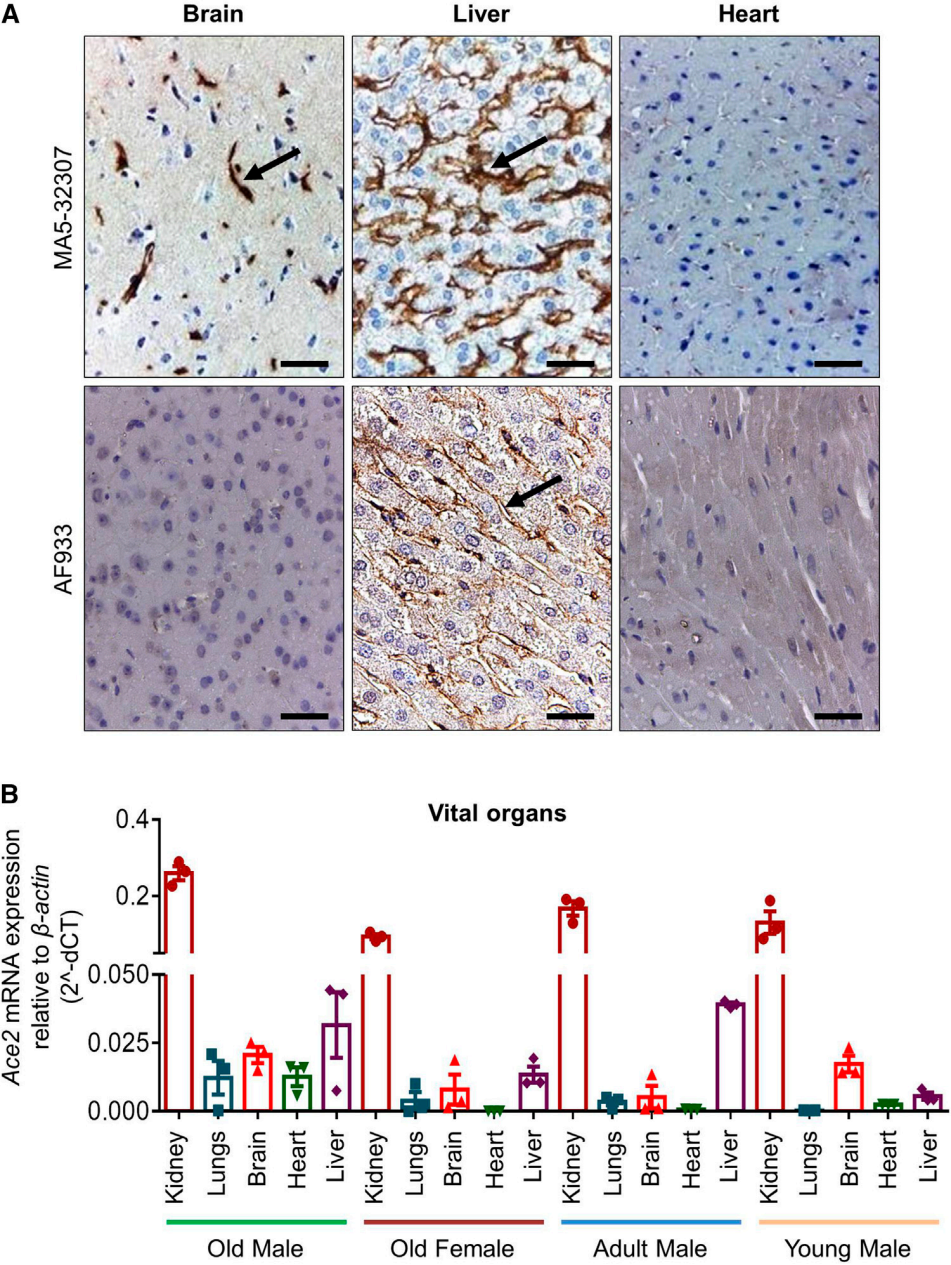
ACE2 expression pattern in hamster brain, liver, and heart. **(A)** Micrographs showing immunohistochemical staining of hamster brain, liver, and heart tissues for ACE2. The arrow symbol indicates positively stained cells in different tissues (scale bar = 50 µm). **(B)** Graph showing *Ace2* relative mRNA expression in hamster vital organs from different age and sex groups (n = 3).

In human patients, the lung associated pathology is the predominant feature of SARS-CoV-2 infection (Jain, 2020). Certain earlier studies have shown the expression of ACE2 transcripts or protein by lung epithelial cells (Lukassen et al., 2020; Hamming et al., 2004; Sungnak et al., 2020; Zhang et al., 2020). Hence, just after the reports that ACE2 binds with SARS-CoV-2 spike (S) protein, the research and clinical communities assumed a high level of ACE2 expression in the lung or other respiratory tract parts might be a major driving factor in the pathogenesis of this respiratory virus. Our initial immunoblot analysis data showed a very trace amount of ACE2 expression in lung tissue lysate (Figure 1A). To get an idea about the spatial and cell-type distribution of ACE2 expressing cells in the lungs and trachea, further IHC analysis was conducted. Interestingly, with both the antibodies (MA5-32307 and AF933), we did not find any visible positive staining in the epithelial cells of the lung bronchioles, and alveoli (Figure 2A). The pattern was consistent irrespective of age and sex of animals (data not shown). Endothelial cells and smooth muscle cells associated with the wall of blood vessels were also negative for ACE2 staining (data not shown). Corroborating our IHC data, IF staining of lung tissue samples did not show any positive staining compared with corresponding without antibody stained tissue sections or antibody and immunogen preadsorption controls (Figure 2A). In certain instances, we noticed some mild staining of bronchial epithelial cells with AF933 Ab; however, a similar pattern was also noticed after antibody preadsorption with the immunogen (Figure 2A). We believe that the trace amount of lung-associated ACE2 detected in immunoblot analysis (Figure 1A) might have come from some non-epithelial cells with low abundance and scattered distribution in the lung parenchyma, whose presence might not be obvious in stained tissue sections. Our findings corroborate multiple earlier reports, including information available at the human protein atlas (https://www.proteinatlas.org/ENSG00000130234-ACE2/tissue/lung), which have shown no or minimal expression of ACE2 in human lung tissues (Hikmet et al., 2020; Hikmet et al., 2020; Fu et al., 2020; Aguiar et al., 2020). Tracheal epithelial cells showed no positive staining with MA5-32307 antibody. However, AF933 showed strong staining of tracheal epithelial cells, which did not get neutralized after preadsorption of the antibody with the corresponding immunogen. Based on these data, we concluded that tracheal epithelial cells do not have ACE2 expression (Figure 2B). We conducted a qPCR analysis to get an idea about *Ace2* mRNA expression level in hamster lung tissues of different age groups. The results shown in Figure 3B clearly show a very minimal expression of *Ace2* in lung tissues of all the animals. We did not find any obvious difference in the expression level of *Ace2* in males of adult and young hamsters (Figure 3B). However, the average expression level of *Ace2* in old age males’ lung tissues was comparatively higher than adult (3.51 fold) or young (37.19 fold) males. Simultaneously, the *Ace2* expression level in old age males’ was higher (3.17 fold) than corresponding females (Figure 3B). Old age and male sex have been identified as independent risk factors for poor prognosis of COVID-19 patients (Grasselli et al., 2020; Williamson et al., 2020; Zhou et al., 2020). Although through our IHC or IF analysis, we failed to detect any expression of ACE2 protein in lung tissues, but very low level of ACE2 expression in lung tissues cannot be totally ruled out. Together, based on our preliminary findings and available reports, we believe that SARS-CoV-2 related lung pathology might be independent or minimally dependent on ACE2 expression status in the lungs, which warrants further investigation. Recent studies have reported the presence of SARS-CoV-2 viral protein in respiratory tract epithelial cells and lungs of infected hamsters (Sia et al., 2020), hence our findings suggest the possible involvement of some other proteins than ACE2 in the entry of SARS-CoV-2 virus into respiratory and lungs cells (Jeffers et al., 2004; To and Lo, 2004).

Out of other vital organs in the brain, mostly the cerebral cortex neurons were positively stained for ACE2 expression by MA5-32307 (Figure 3A). This finding corroborates information available at the human protein atlas (https://www.proteinatlas.org/ENSG00000130234-ACE2/tissue). Expression of ACE2 in hamster brain neuronal cells might help investigate the possible neurological tissue damage due to SARS-CoV-2 infection (Baig et al., 2020). In liver tissues, mostly the sinusoidal endothelial cells stained positive for ACE2, but hepatocytes were negatively stained (Figure 3A and Supplementary Figure S1A). Our immunoblot analysis data also showed ACE2 expression in hamster liver tissues (Figure 1A). The sinusoidal endothelial expression of ACE2 in the hamster does not match the expression of ACE2 pattern reported for human liver (Hamming et al., 2004), and warrants further investigation to understand these contradictory findings. Our analysis, did not notice any positive staining in the bile duct and gall bladder epithelial cells (data not shown).

**FIGURE 4:**
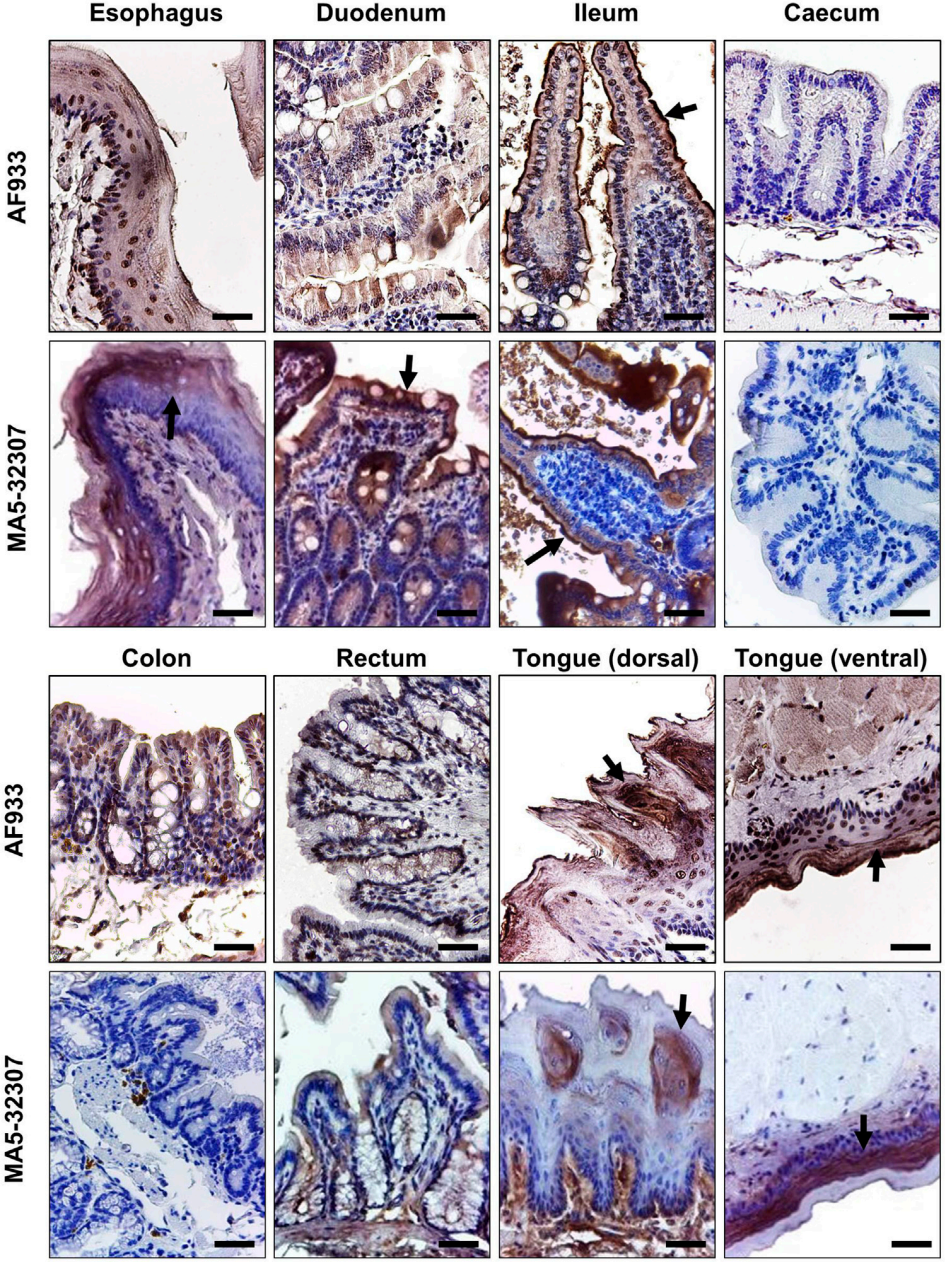
ACE2 expression pattern in different parts of the hamster gastrointestinal tract and tongue tissues. Immunohistochemical analysis showing representative images of ACE2 expression at different parts of the hamster gastrointestinal tract and tongue tissues. The arrow symbol indicates positively stained cells in different tissues (scale bar = 50 µm).

Like ACE2 expression in kidney tissues, we consistently observed a high level of ACE2 expression at different parts of the hamster gut (Figure 4). Prior studies have reported high expression of ACE2 in human gut tissues (Hamming et al., 2004; Hikmet et al., 2020). Single-cell transcriptomic analysis of gut tissues has identified the expression of ACE2 in upper epithelial and gland cells of the esophagus and absorptive enterocytes of ileum and colon (Zhang et al., 2020). Our IHC data shows the expression of ACE2 in the surface epithelial cells of hamster esophagus, duodenum, ileum but no expression in the large intestine (caecum, colon, and rectum) (Figure 4). Although AF933 Ab showed low intense staining of esophagus and duodenum tissues compared to MA5-32307, both the antibodies showed very high surface expression of ACE2 in surface epithelial cells of the ileum. ACE2 expression in enterocytes indicates the possibilities for these cells being infected by SARS- CoV-2 through intestinal routes. Our study and other parts of the world have clearly shown shedding of viral genome in COVID- 19 patients stool samples (Senapati et al., 2020b). Importantly, studies have also reported the isolation of infectious SARS-CoV-2 virus from the stool samples (Amirian, 2020). In the recent past, some elegant studies have clearly shown infection of gut epithelial cells by SARS-CoV-2 (Lamers et al., 2020; Xiao et al., 2020). Diarrhea is a common finding in multiple COVID-19 patients across the world (Liang et al., 2020), and in certain COVID-19 patients, gut epithelial cells damage has also been reported. Hamster model with SARS-CoV-2 infection also showed intestinal epithelial damage and expression of viral protein in enterocytes (Chan et al., 2020a). These data suggest that ACE2 expressed by gut epithelial cells might have a role in the pathogenies of SARS-CoV-2 infection. Considering these points, the hamster might be a suitable model to investigate the intestinal pathogenesis of SARS-CoV-2 infection and evaluate different therapeutics that target ACE2.

The human oral mucosal cavity expresses ACE2, and specifically, this is highly enriched in tongue epithelial cells (Xu et al., 2020). Our IHC study’s data also shows the expression of ACE2 in both the dorsal and ventral stratified squamous epithelium of the hamster tongue. Interestingly, the ventral side epithelial cells have a very high ACE2 expression level than the dorsal side (Figure 4). The absence of ACE2 expression in our immunoblot analysis (Figure 1A) could be due to less proportion of cellular proteins contribution into the total tissue lysate (also a possible reason for a low level of β-actin detection). With both the antibodies, we did not observe any positive staining in hamster spleen tissues (data not shown).

Together, our study has provided a comprehensive idea about ACE2 expression patterns in the hamster’s different tissues. We believe that this information will be instrumental in the optimal use of the Syrian golden hamster as a model for SARS-CoV-2 infection. At the same time, whether the expression of ACE2 in hamsters depends on other pathophysiological or diseased conditions warrants further investigation. In this context, a recent study has demonstrated that ACE2 is an interferon-stimulating gene (ISG), hence at the time of infection or tissue injury, interferon might upregulate ACE2 expression in different organs of hamster (Ziegler et al., 2020). In the future, attempts should also be taken to understand the possible effect of tissue-specific post-translational modification of ACE2 on antibody reactivity in hamster tissues.

## Data Availability Statement

The raw data supporting the conclusions of this article will be made available by the authors, without undue reservation.

## Ethics Statement

The animal study was reviewed and approved by Institutional Animal Ethical Committee (IAEC), Institute of Life Sciences, Bhubaneswar, India.

## Author Contributions

SS conceived the idea, helped in manuscript writing and supervised the study. VS, DP, AM, MS and BSS executed the experiments and helped in manuscript writing.

## Conflict of Interest

The authors declare that the research was conducted in the absence of any commercial or financial relationships that could be construed as a potential conflict of interest.
