# Supplementary material for: Tissue Distribution of ACE2 Protein in Syrian Golden Hamster (Mesocricetus auratus) and Its Possible Implications in SARS-CoV-2 Related Studies
Source: Front Pharmacol. 2021 Jan 14;11:579330. doi: 10.3389/fphar.2020.579330 (PMC7869018; doi:10.3389/fphar.2020.579330)

**Supplementary figure 1:** Immunofluorescence images of hamster tissues stained with antibodies with or without preadsorption.

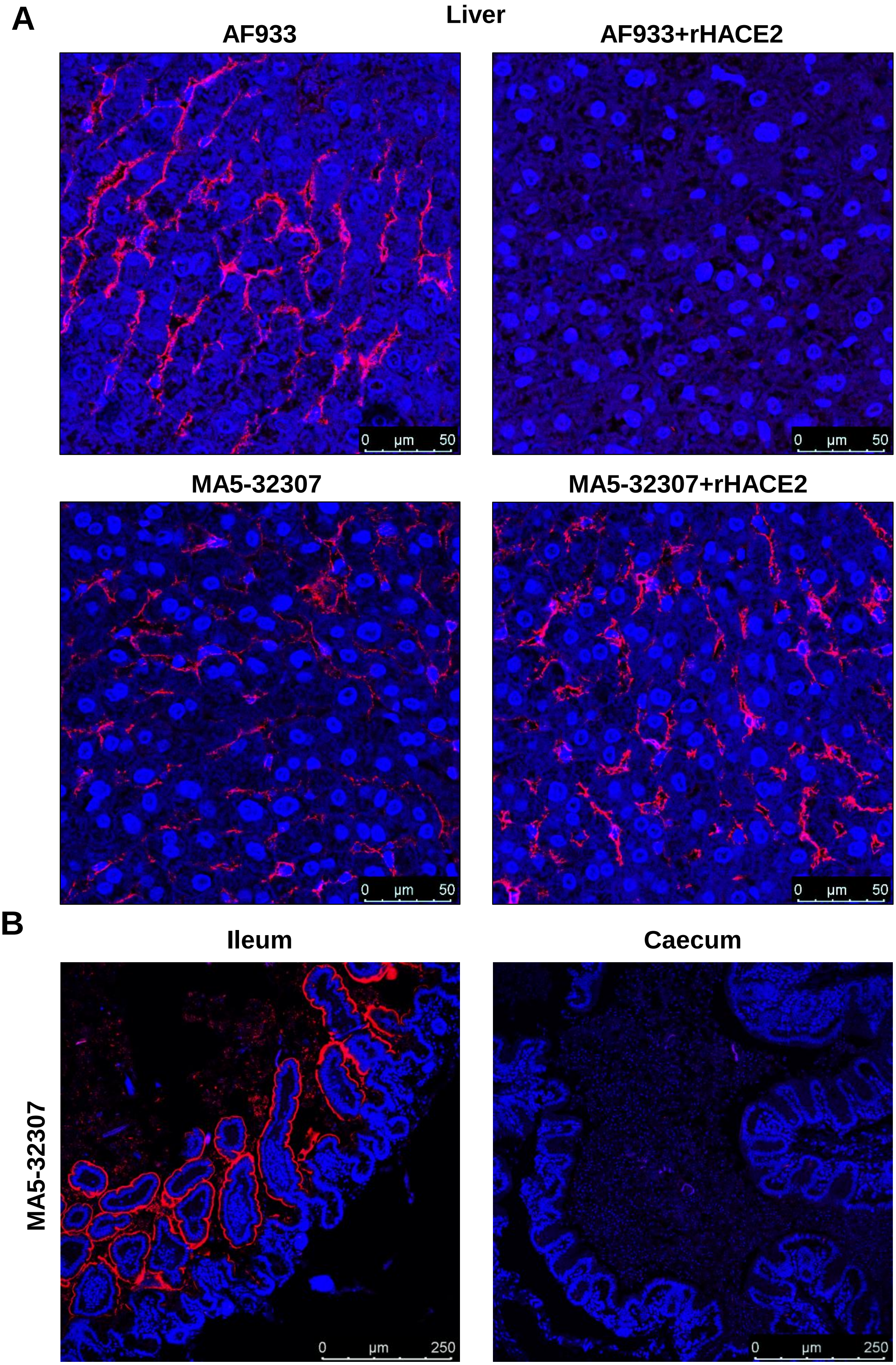

Supplement: Supplementary file 1 [file datasheet1.pdf]
